# Nitrogen Nutrition Improves the Potential of Wheat (*Triticum aestivum* L.) to Alleviate the Effects of Drought Stress during Vegetative Growth Periods

**DOI:** 10.3389/fpls.2016.00981

**Published:** 2016-06-30

**Authors:** Muhammad Abid, Zhongwei Tian, Syed Tahir Ata-Ul-Karim, Yakun Cui, Yang Liu, Rizwan Zahoor, Dong Jiang, Tingbo Dai

**Affiliations:** Key Laboratory of Crop Physiology, Ecology and Production Management, National Engineering and Technology Center for Information Agriculture, Jiangsu Key Laboratory for Information Agriculture, Nanjing Agricultural UniversityNanjing, China

**Keywords:** drought stress, vegetative stages, nitrogen nutrition, photosynthesis, ribulose1, 5-bisphosphate carboxylase/oxygenase, antioxidants, winter wheat

## Abstract

Efficient nitrogen (N) nutrition has the potential to alleviate drought stress in crops by maintaining metabolic activities even at low tissue water potential. This study was aimed to understand the potential of N to minimize the effects of drought stress applied/occur during tillering (Feekes stage 2) and jointing (Feekes stage 6) growth stages of wheat by observing the regulations and limitations of physiological activities, crop growth rate during drought periods as well as final grain yields at maturity. In present study, pot cultured plants of a wheat cultivar Yangmai-16 were exposed to three water levels [severe stress at 35–40% field capacity (FC), moderate stress at 55–60% FC and well-watered at 75–80% FC] under two N rates (0.24 g and 0.16 g/kg soil). The results showed that the plants under severe drought stress accompanied by low N exhibited highly downregulated photosynthesis, and chlorophyll (Chl) fluorescence during the drought stress periods, and showed an accelerated grain filling rate with shortened grain filling duration (GFD) at post-anthesis, and reduced grain yields. Severe drought-stressed plants especially at jointing, exhibited lower Chl and Rubisco contents, lower efficiency of photosystem II and greater grain yield reductions. In contrast, drought-stressed plants under higher N showed tolerance to drought stress by maintaining higher leaf water potential, Chl and Rubisco content; lower lipid peroxidation associated with higher superoxide dismutase and ascorbate peroxidase activities during drought periods. The plants under higher N showed delayed senescence, increased GFD and lower grain yield reductions. The results of the study suggested that higher N nutrition contributed to drought tolerance in wheat by maintaining higher photosynthetic activities and antioxidative defense system during vegetative growth periods.

## Introduction

Plants match their growth rate according to the availability of resources such as light, water, and nitrogen (N) by a number of acclimation mechanisms. Understanding of underlying mechanisms and growth patterns describing how plants respond to optimum and sub-optimum supplies of these resources is essential for developing the appropriate management strategies for enhancing crop productivity and also for improving the resource use efficiency under resource-limited conditions (Teixeira et al., 2014).

Crop productivity is the ultimate product of photosynthesis and inter-linked physiological processes functioning in the plant. Photosynthesis is the main driving force for dry matter (DM) assimilation, organ formation and plant production (Zlatev and Lidon, 2012). Photosynthetic rate and chlorophyll (Chl) fluorescence, and their fluctuation around their normal values are the significant indicators of both plant fitness and extent of environmental stress (Maxwell and Johnson, 2000). By measuring the yield of Chl fluorescence, information about changes in the efficiency of photochemistry and heat dissipation can be obtained (Maxwell and Johnson, 2000). Leaf Chl provides the platform for photosynthetic machinery (Murchie et al., 2009), whereas ribulose-1, 5-bisphosphate carboxylase/oxygenase (Rubisco) is key enzyme involved in the process of photosynthesis (Makino, 2011). Thus, a change in Chl and Rubisco contents under unfavorable conditions may reflect the extent of damage to the light-harvesting complexes of the leaf. Moderate drought stress decreases the photosynthesis mainly due to the stomatal limitations; meanwhile, other interrelated photosynthetic processes are not significantly damaged. In contrast under extreme drought stress when stomatal opening is less than 0.1 mol H_2_O m^-2^s^-1^ then stomatal resistance, poor performance of photosystem II (*F_v_/F_m_*) and downregulated activities of CO_2_ assimilating enzymes such as Rubisco become the dominant limitations to reduced photosynthesis (Grassi and Magnani, 2005).

The limitations in the photosynthetic process as a consequence of intensified drought stress not only imposes direct drought stress-induced damages to plants but also results in light-induced oxidative stress. The lower efficiency of photosynthetic system under drought stress leads to the imbalance between absorbed light energy and its utilization in the carbon assimilation process, which in turn spares more electrons triggering the production of reactive oxygen species (ROS). If the ROS generation is not quenched by antioxidants it causes the protein oxidation, call membrane damage, lipid peroxidation (Saneoka et al., 2004), DNA, RNA and enzymes “oxidative stress,” which impairs the normal growth of the plant (Niu et al., 2013). Plants naturally have been equipped with an elaborate ROS scavenging antioxidant system. Among this system, superoxidase dismutase (SOD) is the front-line antioxidant to catalyze the disproportionation of most reactive superoxide (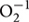) to less reactive hydrogen peroxide (H_2_O_2_) and singlet oxygen (^1^O_2_) (Cruz de Carvalho, 2008). While ascorbate peroxidase (APX) is the key enzyme to catalyze the reduction of H_2_O_2_ into water (Sgherri et al., 2000).

Nitrogen is a vital structural component of proteins, Rubisco, nucleic acids, Chl as well as some hormones and N fertilization is an essential agronomic management practice to enhance the crop productivity (Ata-Ul-Karim et al., 2016). Functional activity of the photosynthetic apparatus of leaf largely depends on the availability of N in plants (Brennan, 1992). In addition, efficient N nutrition has been reported to have the potential to alleviate the drought stress damages by maintaining metabolic activities even at low tissue water potential (Zhang et al., 2007), yet its effects under varying drought stress levels at vegetative growth periods of wheat are not clearly understood. Previous drought stress studies have reported that photosynthetic rate of the leaf under drought stress is closely related to the leaf Chl contents, N concentrations and stay-green characteristics of the leaf, which in turn increases the grain yield by increasing the photosynthetic process (Park and Lee, 2003).

Water limitations accompanied by low N is the main constraint to wheat yield, and has widely been reported to affect the leaf water relations, Chl fluorescence and photosynthetic processes leading to the restricted plant growth rate, early senescence, reduced grain filling duration (GFD) with limited grain weight and poor crop productivity (Park and Lee, 2003; Madani et al., 2010; Mobasser et al., 2014). An adequate assessment of the impacts of drought stress under different N levels on the physiological activities and yield attributes can provide the valuable insights for wheat cultivation under drought stress (Teixeira et al., 2014).

Due to the importance of grain yields, less attention has been focused on studying the effects of drought stress during the vegetative growth periods of wheat. We argue that drought stress study in wheat should be extended to investigate the crop response to drought stress during vegetative growth stages. In wheat, tillering capacity of the crop is a major constituent of the final grain yield (Ata-Ul-Karim et al., 2015), but has been reported to be highly vulnerable to drought stress (Blum et al., 1990). Overall, the vegetative growth period is expected to considerably influence the final grain yield potential in wheat since, the photosynthetic reserves accumulated up to anthesis are estimated to contribute up to 57% of the final grain yield (May, 1976). Similarly, the number of grains, a vital determinant to yield, is also specified by the plant growth rate during stem development and a few weeks before anthesis (Ercoli et al., 2008).

Limited photosynthesis during drought stress might be due to the alterations in N levels and its availability under drought stress conditions (Yang et al., 2012). There is limited information available on this topic, and studies are contradicting. Some studies have reported that N application compensated for the negative effects of drought stress (Jackson et al., 2003; Saneoka et al., 2004; Yang et al., 2012). Morgan (1986) found that N-fertilized wheat plants responded more rapidly to increasing drought stress by closing stomata and reducing net photosynthesis. But Li et al. (2011) mentioned that different grass species under drought stress did not modify physiological functions under varying N application. These inconsistent findings might be attributed to differences in species characteristics, specific environment, N and drought stress levels or diverse growth stages of crops. Even under stable water and N levels, the plant responses to drought stress vary at different growth stages of the crop (Ernst et al., 2010; Shi et al., 2014). Additionally, despite its importance, very few studies provide a complete comparison of the effects of different degrees of drought stress at different vegetative growth stages of wheat on grain yield traits.

The present study was planned to investigate the effects of different water regimes and N levels on physiological activities, growth and yield in wheat, and to find out whether N application improves the potential to withstand and recover from drought stress applied during vegetative growth periods.

## Materials and Methods

### Plant Culture and Growth Conditions

The experiment was carried out in a green house at Pailou Experimental Research Station, Nanjing Agriculture University, China during the growing season of 2014–2015. A winter wheat cultivar Yangmai-16 widely cultivated in the middle and lower Yangtze River Basin was selected as the experimental material for the present study. Before sowing, the wheat seeds were surface-sterilized with 0.5% hypochlorite solution. Fifteen surface sterilized uniform seeds were planted in the free-draining plastic pots having 22 and 25 cm height and diameter, respectively. The pots were filled with 8 kg air-dried and sieved (0.5 mm) clay loam soil having 13% soil moisture, taken from the cultivated field. The soil pH, organic matter, electrical conductivity (EC), bulk density, field capacity (FC) by volume, total N, available phosphorous, and available potassium were 7.6, 1.8%, 0.42 ds/m, 1.29 g cm^-3^, 38.5%, 0.82 g kg^-1^, 19.72 mg kg^-1^ and 78.26 mg kg^-1^, respectively. At the time of soil filling, 0.5 g P_2_O_5_ and 1.1 g K_2_O were applied per pot for each treatment. Thinning was carried out 10 days after germination, and seven uniform seedlings per pot were selected for the subsequent studies. Each pot was irrigated to 80% FC by tap water, characterized by 7.5 pH, 2.8 dsm^-1^ EC and 1200 mg L^-1^ total soluble salts (TSS) until the start of drought stress treatments.

### Drought Stress and Nitrogen Treatments Application and Management

The experiment used three water treatments (severe drought stress, moderate drought stress and well-watering conditions), and two N application rates (0.24 and 0.16 g N/kg soil, designated as N_0.24_ and N_0.16_, respectively). For N treatments, 50% N of each level was distributed at the time of sowing, 30% at jointing and 20% at booting stages, respectively to the each pot. Severe and moderate drought stress treatments to each N level were applied on separate pots during tillering (Feekes 2 stage) and jointing (Feekes 6 stage) wheat growth stages, respectively. At each stage, irrigation to pots was withheld until a moderate drought stress level at 55–60% FC and severe drought stress at 35–40% FC was reached (Cui et al., 2015). These severe and moderate drought stresses at each stage were maintained for 10 days by compensating the lost water. Meanwhile, the well-watered pots were irrigated at 80% FC. After drought stress application, the pots were re-watered to the level of well-watered pots until maturity. Severe and moderate drought stress levels during tillering and jointing stages were designated as ST, MT, SJ, and MJ, respectively while well-watered control pots were designated as WW. Soil water status was measured before water application to the pots. The drought stress was maintained by replenishing the water lost by watering to the desired FC. The amount of water required for irrigation was calculated as:

W=Y×H×A×(FC1−FC0)

where, W is the amount of irrigation water, Y is the soil bulk density, H is the soil depth, A is the area of pot, FC1 is the upper limit of desired soil FC, and FC0 is the actual soil FC before irrigation.

The experiment was laid out in a randomized complete block design with the factorial arrangement. Two N levels (N_0.24_ and N_0.16_), three water regimes (severe, moderate and well-watered) and two growth stages (tillering and jointing) were arranged as first, second and third independent factors, respectively. Thirty pots were allotted to each treatment. The pots with different treatments were rotated on every alternate day to ensure that all the plants received equal radiation and other environmental exposures.

### Plant Sampling and Traits Measurements

Top most fully expanded leaves were sampled from three randomly selected pots (replicates) for each treatment on 1 day before starting drought stress (0DS), 5th and 10th day drought stress (5 DS, 10 DS), and 1and 3 days after re-watering (1 DRW, 3 DRW) to measure leaf water potential (Ψ_w_), membrane stability index (MSI), Chl, protein and Rubisco contents. Meanwhile, gas exchange and Chl fluorescence were also measured. Plants in a single pot were used only once and discarded from the experiment after sampling. All the plant sampling and non-destructive measurements were carried out at 9:00–11:00 h (local time).

#### Leaf Water Potential and Membrane Stability Index Measurements

The leaf water potential (Ψ_w_) was measured according to the method of Bruggink and Huang (1997) using a pressure chamber (PMS Instrument Co., Corvallis, OR, USA). The leaves were firmly fixed in the sealing sleeve of specimen holder of the instrument and pressure was applied until the appearance of sap from the exposed end of the leaf. The reading was noted at this point, which indicated the negative force at which the water was held within the leaf, and expressed as –MPa.

The MSI was measured by using a conductivity meter by following the method of Khanna-Chopra and Selote (2007). Leaf samples of 200 mg were thoroughly washed in double distilled water and placed in 10 mL distilled water with two sets. One set was heated for 30 min at 40°C in a water bath and EC was measured (C1). The second set was boiled for 10 min at 100°C in a boiling water bath and EC was measured (C2). The MSI was estimated by the equation given below:

$$MSI=[1-\left(\frac{C1}{C2}\right)]\times 100$$

#### Chlorophyll, Protein and Rubisco Contents Measurements

Leaf samples of 0.2 g were placed in a vial with 4 mL of dimethyl sulphoxide for pigment extraction to determine Chl content (Bruggink and Huang, 1997). The samples were centrifuged at 5,000 × *g* for 15 min at 4°C to get the supernatant. Then absorbance of the supernatant was measured using a spectrophotometer at 470 and 648 nm to calculate Chl a and b concentration, respectively. The sum of Chl a and b was considered as total Chl contents.

To determine the total soluble protein contents, leaf samples (0.5 g) were ground and extracted in a buffer of sodium phosphate (50 mM, pH 7.0). The mixtures were centrifuged at 4000 × *g* and 4°C for 10 min. To quantify soluble proteins the supernatants of samples were standardized using bovine serum albumin and absorbance values were recorded spectrometrically at 595 nm.

Rubisco content was measured with Western-blot analysis by using the SDS-PAGE method according to the method given by Makino et al. (1985). Leaf samples (0.5 g) were homogenized in a 50 mM Tris–HCL buffer (pH 8.0) containing 12.5% (v/v) glycerol and 5 mM β-mercaptoethanol, and centrifuged for 15 min at 15,000 × *g*. SDS, β-mercaptoethanol, and glycerol with final concentrations of 1% (w/v), 2% (v/v), and 5% (v/v), respectively were added to the supernatant and the mixture was heated for 5 min. A 10 μL sample of this prepared solution was used for electrophoresis. The gels were stained with 0.1% (w/v) Coomassie Brilliant Blue R-250 solution. The stained bands from the gels were excised and eluted in 1 mL of formamide at room temperature with agitation for 8 h with an unstained gel as a standard. The blots were finally washed three times in phosphate-buffered solution as above and developed with Super Sigmal West Pico Chemiluminescent Substrate (Pierce, USA). Images of the blots were scanned using a CCD imaging system (Fluor SMax, Bio-Rad, USA) and Quantity One software (Bio-Rad, Hercules, CA, USA) was used to calculate the optical density.

#### Leaf Gas Exchange Measurements

Leaf gas exchange measurements were carried out on the uppermost fully developed leaves using an open system (LI-6400 Inc., Lincoln, NE, USA). Photosynthetic measurements were made under a light level of 1000 μmol photon m^-2^ s^-1^, 25°C and 400 μmol mol^-1^ of CO_2_ (Ca). The light-saturated net CO_2_ assimilation rate (*P_n_*) and stomatal conductance (*g_s_*) were measured.

#### Chlorophyll Fluorescence Measurements

Chlorophyll fluorescence was determined using a modulated fluorometer (FMS2; Hansatech, King’s Lynn, Norfolk, UK). Before measurements, top fully expanded leaf blades of evenly oriented and maximum light-exposed leaves were dark adapted for 30 min, and ground-state fluorescence (*F0*) and maximal fluorescence (*Fm*) were recorded. Then steady-state fluorescence values (*Fs*) and maximal fluorescence of light-adapted level (*Fm′*) were measured. Next, the actinic light was turned off and the minimal fluorescence in the light-adapted state (*F0′*) was determined by illuminating the leaf with far-red light for 3 s. From these base values, the fluorescence and heat dissipation parameters as the maximum efficiency of PS II (*Fv/Fm*), effective quantum yield of PS II (Φ_PSII_) and non-photochemical quenching (NPQ) were calculated as proposed by Maxwell and Johnson (2000).

#### Antioxidants Activities and Lipid Peroxidation Determination

The activities of superoxide dismutase (SOD), APX and lipid peroxidation [estimated by measuring the malondialdehyde concentration (MDA)] were determined according to the methods given by Jiang and Zhang (2002). Frozen leaf samples of 0.5 g were sliced and homogenized in a mortar, and pestle with 5 mL ice-cold extraction buffer containing 50 mM potassium phosphate buffer (pH 7.0) and 0.4% polyvinyl poly pyrrolidone (PVP). The homogenates were centrifuged at 10,000 × *g* for 30 min at 4°C and the supernatant was used as crude extract for the above assays.

Superoxide dismutase (SOD) activity was determined by adding 0.1 mL enzyme extract to a reaction mixture of 1.5 mL 50 mM sodium phosphate (pH 7.8), 0.3 mL 130 μM methionine, 0.3 mL 750 μM nitro-blue tetrazolium (NBT), 0.3 mL 100 μM EDTA-Na_2_, 0.300 mL 20 μM riboflavin and 100 μL distilled water, and illuminated in light of 4000 flux for 20 min and the sample absorbance was determined at 560 nm wavelength by using a Pharmacia Ultra Spec Pro UV/VIS spectrophotometer (Pharmacia, Cambridge, England). One unit of SOD activity was considered as the amount of enzyme used for 50% inhibition of the NBT reduction.

For APX activity determination, 0.2 mL enzyme extract was added to a reaction mixture of 50 mmol L^-1^ potassium phosphate buffer (pH 7.0), 0.5 mmol L^-1^ ASC and 0.1 mmol L^-1^ H_2_O_2_. APX activity was determined by observing the decrease at 290 nm wavelength for 1 min in 1 mL of the reaction mixture.

Malondialdehyde (MDA) determined through thiobarbituric acid (TBA) analysis. MDA concentration was measured at 532 nm wavelength and corrected by subtracting the absorbance at 600 nm wavelength by using an extinction coefficient of 156 mmol L^-1^ cm^-1^ by the equation; MDA (mmol g^-1^FW) = [(A532 – A600)/156] × 103 × dilution factor.

#### Relative Growth Rate and Dry Matter Accumulation Determination

The whole plants from three randomly selected pots for each treatment were manually cut at ground level using pruning-scissors at tillering, jointing, anthesis and maturity to weigh the fresh weights. Samples were oven-dried at 105°C for 30 min and then at 70°C until constant weight to obtain DM (g/pot).

The relative growth rate (RGR) was calculated as the rate of DM accumulation per unit of existing DM between the two adjacent sampling/growth stages.

$$RGR=\frac{1}{DM}\times \frac{\Delta DM}{\Delta d}$$

where, DM is the DM of the sampling stage, ΔDM is the change in DM between two adjacent sampling/growth stages and Δd is the number of days between two sampling stages.

The pre-drought limitation (PDL) of drought treatments for DM production at maturity was estimated according to Xu et al. (2009):

$$PDL(\%)=\frac{\left(DMC-DMT\right)}{DMC}\times 100$$

where, DMC is total DM in pots under well-watered conditions, which were not exposed drought-stressed throughout the experimental period, while DMT is the total DM in pots which were drought-stressed.

#### Crop Phenology, Grain Filling Rate and Grain Yield Components Determination

The number of days from planting to anthesis was recorded when plants under a treatment had completed 50% anthesis. Similarly, GFD (days from anthesis to grain maturity) were recorded when 50% of spikes under a treatment had reached grain maturity. Grain filling rate (GFR) was calculated by randomly selecting five spikes from each treatment starting from 7 days after anthesis (DAA) with the interval of 7 days until completion of grain filling phase. Ten grains from the middle part of each spike were taken and oven dried at 70°C to a constant weight. Then GFR (mg/grain/day) was noted as the increase in grain dry weight per unit time.

At maturity, three pots were randomly selected from each treatment to count the number of tillers bearing spikes, number of grains/spike, 1000-grain weight and grain yield/pot.

Drought index (DI) was calculated as grain yield differences between drought stress and WW growing conditions (Zhang et al., 2007).

$$DI=\frac{YD}{YW}$$

where, YD is the grain yield under drought stress condition and YW is the grain yield under WW conditions.

### Statistical Analysis

The analysis of variance (ANOVA) was performed using the General Linear Model procedure to calculate the effects of drought stress and N levels on the morphological and physiological parameters at each sampling and measurement point. Means were compared using Duncan’s multiple comparison tests (*p* < 0.05) (SPSS Inc., Chicago, IL, USA).

## Results

### Leaf Water Potential and Membrane Stability Index

**FIGURE 1:**
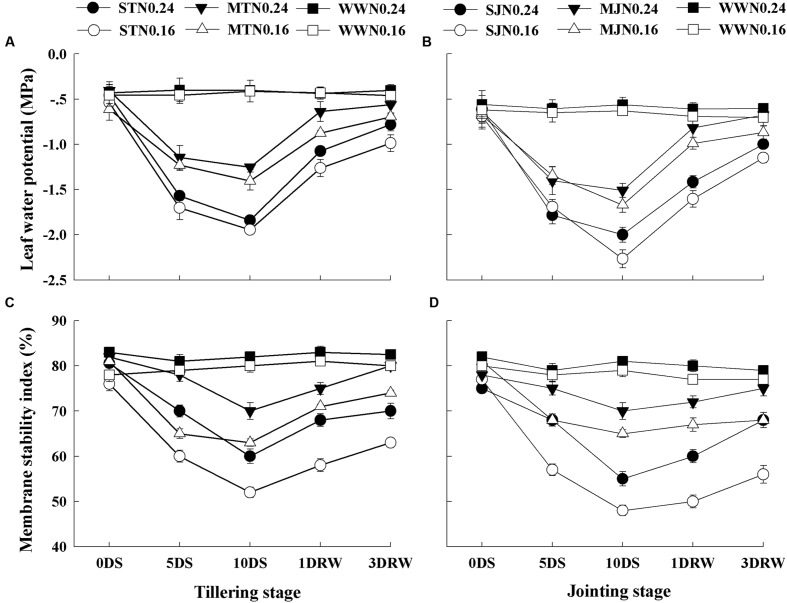
**Leaf water potential **(A,B)** and membrane stability index **(C,D)** as affected by severe and moderate drought stress applied at tillering and jointing under two N rates.** ST, MT, SJ, and MJ were designated for severe and moderate drought stress during tillering and jointing stages, respectively and WW for well-watered treatment. Time-course of the measurements was one day before stress (0 DS), 5th and 10th day of stress (5 DS, 10 DS), 1 and 3 days after re-watering (1 DRW, 3 DRW).

There was a progressive decline in the Ψ_w_ and MSI during the drought stress (**Figure 1**). There was no significant difference in Ψ_w_ and MSI between the two N levels under WW conditions, but there was a significant difference between treatments on these parameters under drought stress conditions. Ψ_w_ and MSI declined more under low N application and greater decline was observed at jointing stage as comapred to the tillering stage under drought stress. After re-watering, the Ψ_w_ and MSI tended to restore differently under drought stress and N rates. The drought-stress plants showed greater recovery in Ψ_w_ and MSI under N_0.24_ as compared to N_0.16_.

### Chlorophyll, Soluble Protein and Rubisco Contents

**FIGURE 2:**
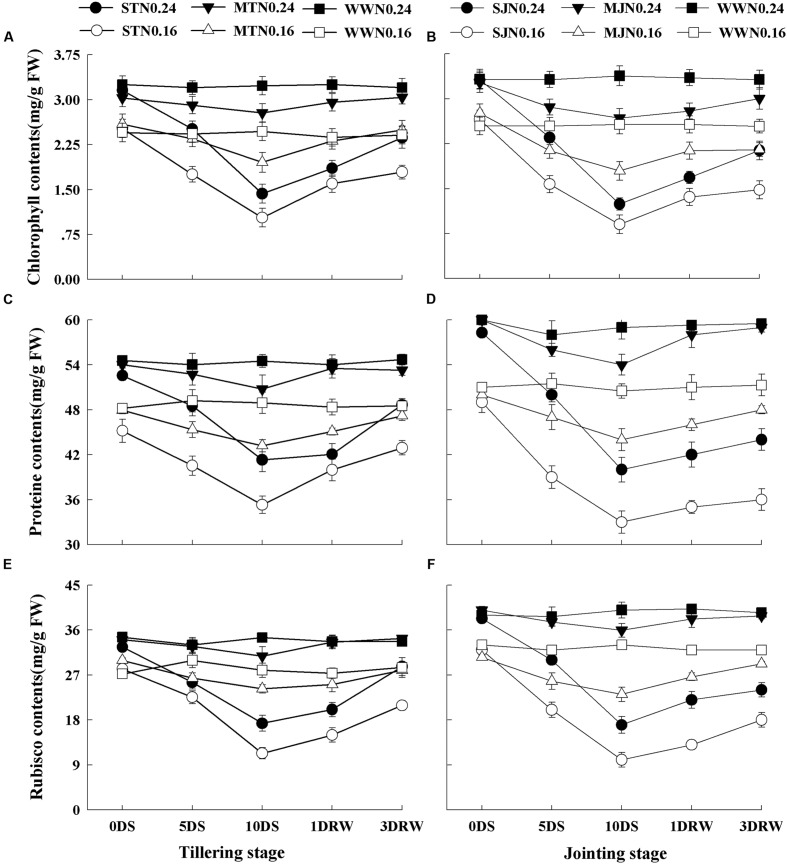
**Contents of chlorophyll **(A,B)**, protein **(C,D)** and Rubisco **(E,F)** as affected by severe and moderate drought stress applied at tillering and jointing under two N rates.** ST, MT, SJ, and MJ were designated for severe and moderate drought stress during tillering and jointing stages, respectively and WW for well-watered treatment. Time-course of the measurements was 1 day before stress (0 DS), 5th and 10th day of stress (5 DS, 10 DS), 1 and 3 days after re-watering (1 DRW, 3 DRW).

The Chl, soluble protein and Rubisco contents were significantly affected under drought stress treatments (**Figure 2**), depending upon the intensity of drought stress and N application rates (*p* < 0.05). The plants under N_0.16_ showed lower values of Chl, protein and Rubisco contents as compared to N_0.24_ under both drought stress and WW conditions. Under drought stress, MT and MJ did not significantly decrease the Chl, protein and Rubisco consents under N_0.24_, while this decrease was significant under N_0.16_. After re-watering, slower recovery of Chl, protein and Rubisco contents was observed in ST and SJ plants at both N levels, however, comparatively greater recovery was observed under N_0.24_.

### Leaf Gas Exchange

**FIGURE 3:**
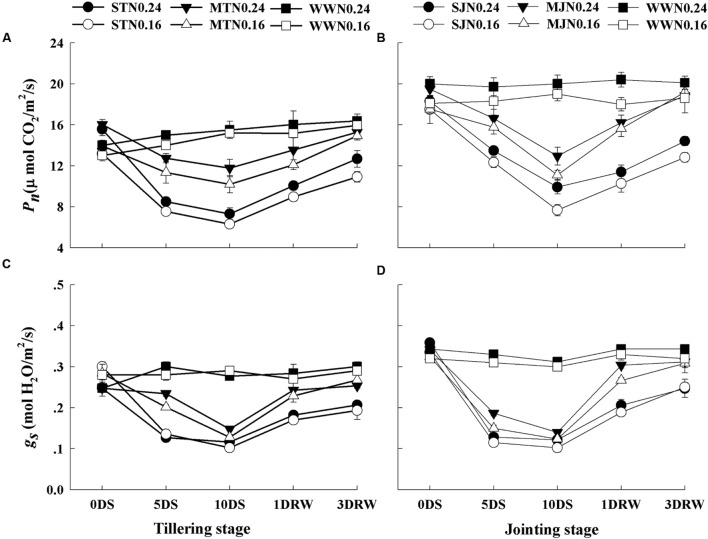
**Net photosynthetic rate (*P_n_*; **A,B)** and stomatal conductance (*g_s_*; **C,D)** as affected by severe and moderate drought stress applied at tillering and jointing under two N rates.** ST, MT, SJ, and MJ were designated for severe and moderate drought stress during tillering and jointing stages, respectively and WW for well-watered treatment. Time-course of the measurements was 1 day before stress (0DS), 5th and 10th day of stress (5 DS, 10 DS), 1 and 3 days after re-watering (1DRW, 3DRW).

Under MT, MJ and WW conditions, lower *P_n_* was observed under N_0.16_ than N_0.24_, however, *g_s_* was not significantly affected by N treatments (**Figure 3**). The *P_n_* and *g_s_* were affected under severe drought stress treatments, depending upon N application rates. *P_n_* and *g_s_* were downregulated under severe and moderate stress and greater down-regulation was observed under N_0.16_ treatment. ST and SJ showed a significant decrease in *P_n_* and *g_s_* and this decline was higher under SJ. After re-watering the stress treatments, complete recovery for *P_n_* and *g_s_* was observed while comparatively slower recovery was observed under N_0.16_ as compared to N_0.24_.

### Chlorophyll Fluorescence

**FIGURE 4:**
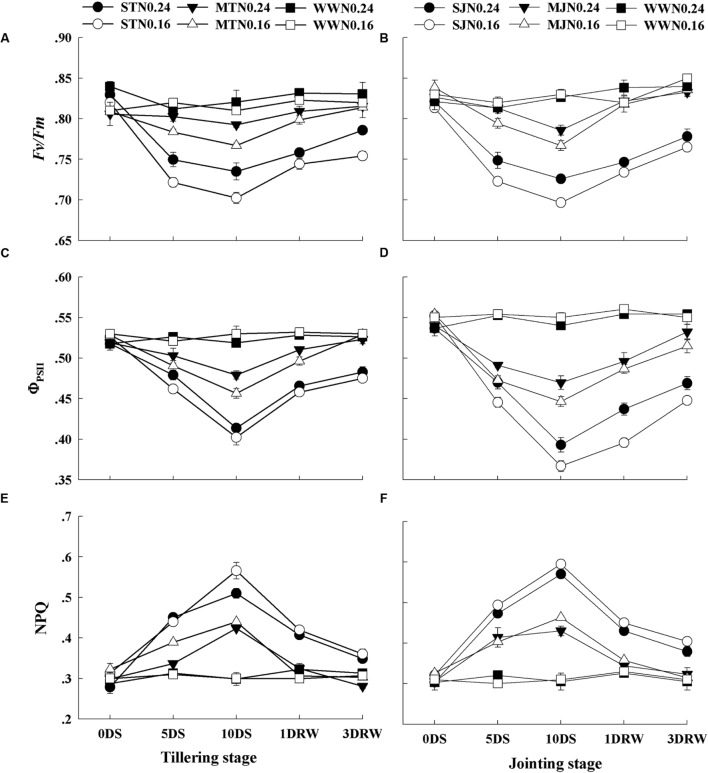
**Quantum efficiency of the photochemical reaction in PSII (*Fv/Fm*; **A,B)**, the quantum yield of the PSII electron transport (Φ_PSII_; **C,D)**, and the heat dissipation through non-photochemical quenching (NPQ; **E,F)** as affected by severe and moderate drought stress applied at tillering and jointing under two N rates.** ST, MT, SJ, and MJ were designated for severe and moderate drought stress during tillering and jointing stages, respectively and WW for well-watered treatment. Time-course of the measurements was 1 day before stress (0 DS), 5th and 10th day of stress (5 DS, 10 DS), 1 and 3 days after re-watering (1DRW, 3DRW).

There was no significant difference in *Fv/Fm*, Φ_PSII_ and NPQ values between N_0.16_ and N_0.24_ under WW conditions (**Figure 4**). *F_v_/F_m_* did not decrease under moderate stress on 5DS, however, but a minor decrease was observed with the progression of stress to 10DS. In contrast, severe stress showed significantly lower *F_v_/F_m_* values under N_0.16_ than that N_0.24_. The Φ_PSII_ values in drought-stress plant also deviated from the values of WW under drought stress and N treatments. The decrease in Φ_PSII_ was greater in JS under N_0.16_ than N_0.24_. Higher NPQ values were recorded in plants under drought stress as compared to those under WW and this increase was pronounced with increasing drought stress. Moreover, the plants under N_0.16_ showed greater NPQ values than N_0.24_ and these values were higher at jointing stage than the tillering stage. After re-watering, *Fv/Fm* and Φ_PSII_ increased while NPQ decreased. The plants under severe stress showed in-complete recovery after re-watering, especially under N_0.16_ application.

### Lipid Peroxidation and Antioxidants Activities

**FIGURE 5:**
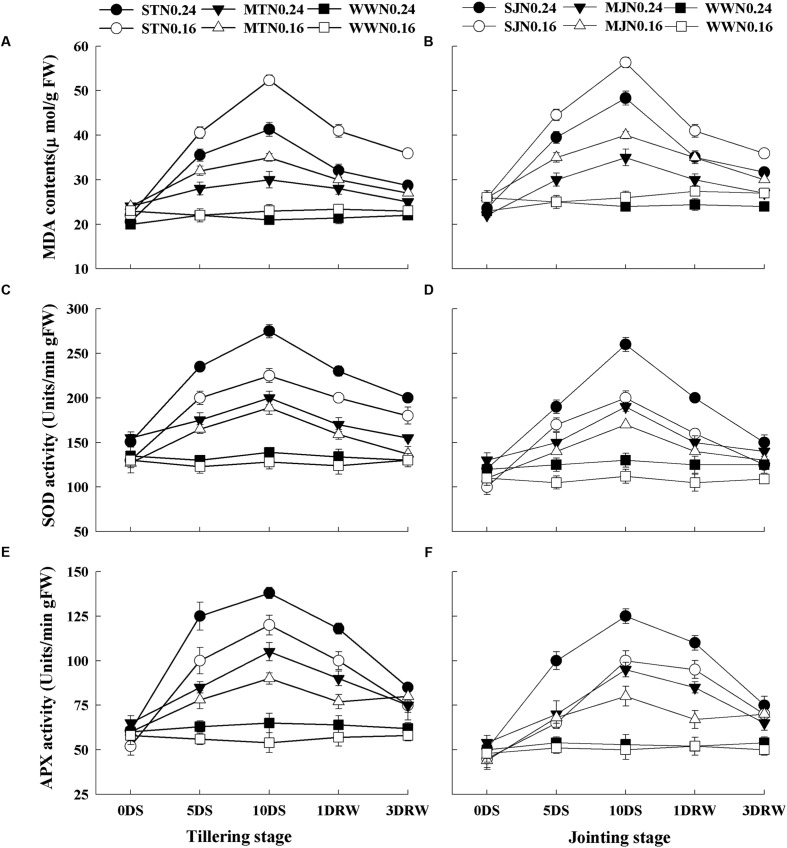
**Malondialdehyde contents (MDA; **A,B)**, superoxide dismutase (SOD; **C,D)** and ascorbate peroxidase (APX; **E,F)** activities as affected by severe and moderate drought stress applied at tillering and jointing under two N rates.** ST, MT, SJ, and MJ were designated for severe and moderate drought stress during tillering and jointing stages, respectively and WW for well-watered treatment. Time-course of the measurements was 1 day before stress (0 DS), 5th and 10th day of stress (5 DS, 10 DS), 1 and 3 days after re-watering (1 DRW, 3 DRW).

Lipid peroxidation estimated by MDA, SOD and APX activities were significantly higher under drought stress treatments as compared to WW treatment (**Figure 5**). There were no significant differences in SOD and APX activities as well as MDA contents between N levels under WW conditions. In contrast, a decrease in MDA content and an increase in SOD and APX activities were observed with increasing N application rate in the drought-stress plants. After re-watering, MDA contents, SOD and APX activities were decreased in drought-stress plants. The lower decrease in MDA contents was observed in N_0.16_ plants under severe stress.

### Relative Growth Rate and Dry Matter Production

Drought stress applied during vegetative growth stages in the present study resulted in a significant reduction in RGR and DM production (**Table 1**). RGR was less affected by N levels as compared to drought stress treatments. The decline in RGR was less from tillering to jointing, while from jointing to anthesis these effects were greater under drought stress as compared to WW conditions. DM productions also showed similar declining trends under different drought stress levels. Maximum decline in DM production was observed in ST (30%) and SJ (36.4%) compared to WW treatments. The impact of drought stress on DM production was less under N_0.24_as compared to N_0.16_. Higher effect of drought stress on DM production under low N application was also indicated by greater PDL values under N_0.16_. The PDL values (21, 12, 31, and 22%) under ST, MT, SJ and MJ treatments, respectively were greater under N_0.16_ than those (18, 8, 29, and 11%) under N_0.24_.

**Table 1:** Effects of drought stress under two nitrogen rates on dry matter production and relative growth rate growth rate during growth periods of wheat.

		Relative growth rate (mg/g/day)			Dry matter production (g/pot)				Pre-droughtlimitation (%)
				
Nitrogen rates	Water regimes	Tillering–jointing	Jointing–anthesis	Anthesis–maturity	Tillering	Jointing	Anthesis	Maturity	Maturity
N_0.24_	ST	10.69d	22.78b	12.21c	5.65d	14.78c	67.02c	102.63c	18.7c
	MT	11.94c	26.03a	15.06ab	6.38c	20.8ab	80.20b	118.77ab	7.6e
	SJ	13.73ab	20.41c	11.54d	8.79a	12.47d	61.69d	87.5d	29.1a
	MJ	14.98a	23.66b	14.49b	8.81a	17.97b	69.52c	109.53b	11.2d
	WW	13.73ab	25.84a	15.68a	8.68a	22.23a	85.27a	132.3a	0.00
N_0.16_	ST	9.55f	21.9bc	12.54c	5.02d	11.42d	53.82e	85.6d	21.5b
	MT	10.7e	22.84b	13.69b	5.71b	14.4c	58.55d	92.3d	12.3d
	SJ	12.88b	18.31d	10.7e	7.27b	9.66e	50.6d	82.23d	31.8a
	MJ	13.15b	20.06c	12.25c	7.96b	13.13c	55.11e	91.6d	21.9b
	WW	12.94b	26.35a	14.6b	7.65b	18.99b	66.12c	105.17c	0.00


*Different lowercase letters following the data within the same column indicate significant differences at *p* < 0.05.*

### Crop Phenology and Grain Filling Patterns

Drought stress and N treatments significantly altered the number of days from planting to anthesis and GFD (**Table 2**). However, moderate stress had little effect on these parameters. Severe drought stress shortened the days from planting to anthesis and GFD at both N levels. Irrespective of drought stress treatments, low N reduced days to anthesis and GFD. The low N strengthened the effect of drought stress on these traits, which was more pronounced at jointing stage stress than that tillering. The lowest days to anthesis and GFD were observed under SJ under N_0.16_ (160.7 and 33.3) and N_0.24_ (163.1 and 34.9).

**Table 2:** Effects of drought stress under two nitrogen rates on crop phenology and grain filling rate of wheat.

Nitrogen rates	Water regimes	Days to anthesis	Grain filling duration	Grain filling rate (mg/grain/day)				
				(DAA)				
				
				7–14	14–21	21–28	28–35	35-maturity
N_0.24_	ST	164.2e	37.3bc	0.79d	2.11c	2.06b	1.38d	0.57c
	MT	172.8ab	38.7b	0.76d	1.84e	2.29a	2.18a	0.89a
	SJ	163.7e	34.1d	0.85c	2.14c	1.85c	1.23d	0.14d
	MJ	171.5b	37.5bc	0.73e	1.84e	2.28a	2.09b	0.78b
	WW	173.7a	40.9a	0.77d	1.88e	2.33a	2.14a	0.93a
N_0.16_	ST	162.8ef	35.91c	0.90b	2.32b	1.69c	1.11d	0.17d
	MT	169.5c	36.18c	0.78d	2.05d	2.13b	1.82c	0.67c
	SJ	160.7f	33.0e	0.95a	2.46a	1.55d	0.82e	0.00e
	MJ	167.1d	35.04c	0.77d	2.03d	2.09b	1.70c	0.61c
	WW	170.3bc	38.31b	0.86c	2.15c	2.31a	1.98b	0.82b


*Different lowercase letters following the data within the same column under each N level indicate significant differences at *p* < 0.05.*

The effect of drought stress on GFR also varied under both levels of N and growth stages at which drought stress was applied. Higher GFR during early grain filling period (7–21 DAA) and lower GFR during later grain filling period (21–42 DAA) were recorded under severe drought stress and low N as comapred to moderate drought stress and WW treatments at high N level.

### Grain Yield Traits

The results showed that the grain yield, number of spikes/pot, number of grains/spike and 1000-grain weight responded to drought stress variably depending upon the drought stress intensity, growth stage and N application rates (**Table 3**). The magnitude of grain yield reduction increased with increasing drought stress intensity and N deficiency. Yield penalties were higher at jointing stage stress than at tillering stage. The extent of grain yield limitation under ST, MT, SJ and MJ was 24.8, 7.4, 31.5 and 15.7% under N_0.16_, whereas under N_0.24_, it was 15.9, 2.3, 23.8 and 5.8%, respectively, compared to WW conditions. The lower grain yield decrease under N_0.24_ resulted in greater DI for drought stress treatments. Significantly higher DI was recorded in MT (0.98 and 0.91) and MJ (0.90, 0.83) under N_0.24_ and N_0.16_, respectively, and lower trends of DI were exhibited by ST (0.84 and 0.81) and SJ (0.76 and 0.0.73) under N_0.24_ and N_0.16_, respectively.

**Table 3:** Effects of drought stress under two nitrogen rates on grain yield traits of wheat.

Nitrogen rates	Water regimes	Grain yield (g/pot)	Drought index	Spikes/pot	Grains/spike	1000-grainWT(g)
N_0.24_	ST	43.01c	0.84d	26.62c	40.11cd	37.09b
	MT	49.73a	0.98a	31.66ab	43.39ab	39.26a
	SJ	38.94d	0.76e	30.66b	33.94f	32.97d
	MJ	46.62b	0.89c	31.12ab	41.96c	36.11c
	WW	51.13a	0.00	32.66a	44.84a	38.32ab
N_0.16_	ST	31.18f	0.82d	18.1f	39.53d	34.42cd
	MT	38.88d	0.91b	23.33d	40.76cd	35.76c
	SJ	29.77f	0.73f	20.33e	34.72f	30.67e
	MJ	35.41e	0.83d	24.73cd	37.36e	34.05cd
	WW	42.98c	0.00	26.66c	42.45b	37.82b


*Different lowercase letters following the data within the same column under each N level indicate significant differences at *p* < 0.05.*

The effects of drought stress treatments and N levels on grain yield were attributed to their parallel effects on spikes/pot especially at tillering, and on grains/spike and 1000-grain weight particularly under jointing stage drought stress (**Table 3**). N_0.24_ significantly increased the number of spikes under drought stress as well as WW treatments. The number of spikes was decreased under ST, MT, SJ, and MJ by 28.2, 8.5, 20.0, and 3.3% at N_0.16_, whereas at N_0.24_ these were decreased by 18.5, 2.1, 6.0, and 2.6%, respectively, compared to WW treatments. Under WW conditions, minor differences in grains/spike were observed between N levels. However, under, ST, MT, SJ, and MJ, the number of grains per spike were decreased by 16.3, 8.5, 27.6, and 12.3%, and by 15.5, 3.1, 22.3, and 8.9% under N_0.16_ and N_0.24_, respectively as compared to WW treatments. The 1000-grain weight was also affected by drought stress and N levels. It was not significantly reduced under MT and ST at N_0.24_, however, under N_0.16_, all stress treatments ST, MT, SJ, and MJ gave significantly lower 1000-grain weight compared to WW pots.

## Discussion

The ability of wheat plants to withstand the drought stress at tillering and jointing stages varied with the intensity of drought stress and N availability. The immediate response of plants to drought stress was observed by decreased Ψ_w_ declines leading to stomatal closure and reduced photosynthesis. The maintenance of Ψ_w_ under higher N application allowed plants to maintain leaf processes functioning during drought stress conditions and to recover faster after the drought ended. Wheat plants given higher N treatment showed higher stomatal activity and membrane stability at a given level of drought stress compared to the plants given low N fertilization. The inhibited growth in N_0.16_ plants under drought stress might be associated to the decreased Ψ_w_, which could result in limited cell growth and development. Limited Ψ_w_ under abiotic stresses is the main cause of loss in DM production and grain yield (Gepstein and Glick, 2013).

The decline in Chl during drought stress accelerated a reduction in the photochemical activities of chloroplasts, which were responsible for the decrease in photosynthesis. The major consequence of drought stress accompanied by limited N availability was reflected in greater degradation of Chl and Rubisco contents. A major part of plant N is stored in the enzymes participating in the photosynthesis especially Rubisco, which is a key source of N recycling. Similar effects of N limitation on Rubisco concentration and photosynthesis have also previously been reported by Gonzalez-Real and Baille (2000) and Makino (2011). Under severe stress, carboxylation efficiency of Rubisco is greatly diminished and it acts more as oxygenase than carboxylase decreasing the photosynthetic carbon assimilation (Makino, 2011). N being an important component of Chl, proteins and Rubisco affected the whole metabolism of the plant during drought stress. Furthermore, Grassi and Magnani (2005) suggested that sufficient N enhances the recovery of photosynthetic biochemistry, and its limited recovery is observed in plants subjected to severe drought stress under limited N. Thus in this study, reduced N level in drought stress treatments resulted in a decreased concentration of Rubisco, and its impairment might lead to an increase in photo-inhibition by decreasing the photosynthetic energy (ATP) consumption in the Calvin Cycle due to decreased electron transport rate. Sufficient N availability might improve photosynthetic capacity and stomatal control in water and N deficit conditions because more than half of the N in the crop green area is active in collecting light to drive the photosynthesis (Sinclair and Jamieson, 2006). Hence, the rate of photosynthesis per unit leaf area can be enhanced by increasing the total amount of photosynthetic pigment (Chl) per unit leaf area in the presence of an optimal N concentration.

As the drought stress intensified from moderate to a severe level, the transpiration rate decreased, stomata were closed and the amount of heat dissipation was increased as identified by greater NPQ values. Greater NPQ values also indicate the protection of photosynthetic apparatus from photo-induced damage under high light intensity because excess excitation energy was required to be safely removed through heat dissipation to prevent the generation of ROS. This phenomenon also assists a rapid and maximum photosystem recovery after re-watering (Gallé et al., 2007). Therefore, maintenance of plant’s water status and stomatal opening under high N were important to maintain the leaf conductance for CO_2_, photosynthetic reactions and electron transport (Liu et al., 2013).

The extent of MDA accumulation in leaves was dependent on the severity of drought stress and N treatments. Severe drought stress under low N resulted in excessive accumulation of MDA as a by-product of respiratory metabolism. MDA damage might trigger multiple developmental signals characterized by the loss of Chl, increased cell membrane permeability, breakdown of macromolecules and remobilization of nutrients as well as early senescence, which ultimately reduced the grain development period. Higher MDA content observed under low N during drought stress indicated the suppressed capacity of sub-cellular ROS scavenging antioxidant system and higher accumulation of ROS in low N plants. In addition, the inhibition of photosynthesis process and the predominance of photo-respiration also lead to the accumulation of ROS in the cell organelles (Galmés et al., 2007). A tight control of ROS generation and accumulation in the plant cell is essential because the over-produced ROS can cause cell death, reducing the plant growth and productivity (Bieker and Zentgraf, 2013). The lower MDA contents in high N plants as compared to low N plants indicated their improved ROS scavenging capability under drought stress conditions.

Greater SOD and APX activities as well also lower MDA content identified in given high N fertilization plants indicated their improved redox defense status to scavenge ROS damage. The development of favorable ROS detoxifying antioxidant system in the drought-stressed plants under higher N in this study might have contributed toward the protection of the photosynthetic process and these findings were in consensus with Saneoka et al. (2004), who suggested that N nutrition contributed to the drought tolerance in bent grass by preventing the cell membrane damage, lowering MDA accumulation, as well as improving osmoregulation.

The severe drought stress during vegetative stages due to its higher interference with plants physiological processes resulted in poor crop establishment, growth rate and final productivity of wheat plants. RGR was decreased by drought stress more under low N fertilization associated with greater decreases in photosynthesis during drought the period and lower photosynthetic recovery after re-watering. When *P_n_* is inhibited, the photosynthetic reserves of the plants are depleted due to continuous respiration (Abid et al., 2016). The imbalance between photosynthesis and respiration is likely to decrease the net plant carbon balance in the drought-stressed plants, which may partially why growth rate declined under drought stress (Galmés et al., 2007). When drought-stressed plants were re-watered, the RGR was recovered almost to the level of WW plants during the next growth periods, which suggests the reversibility of the physiological fluctuations after re-watering from the downregulated activities under drought stress. Although after re-watering, the RGR was restored to WW plants, but the DM accumulation at maturity could not reach the level of DM production under WW treatments under both N levels indicating the existence of PDLs.

To avoid the reproductive failure under severe drought stress, the plants exhibited phenological changes by undergoing earlier anthesis and maturity. Altered crop phenology under stress is an important indicator for sustaining the grain yield in cereals (Chaves et al., 2002). Drought stress under both levels of N accelerated GFR with reduced GFD which in turn reduced the number of grain/spike and grain weights. These results were in consensus with the findings of Wu et al. (2011), in which they reported that drought stress during the vegetative growth period affected wheat crop establishment and hampered crop production under drought stress and low N supply conditions. In our study, the plants with high N availability showed their ability to set a more number of grains as well as to sustain a higher rate of carbon accumulation through photosynthesis in relatively long lasting canopy during grain filling period. In contrast, the drought-stressed plants under low N supply produced a fewer number of grains and the small canopy that had lower photosynthetic carbon supply. **Table 4**, shows that varying drought stress and N levels were the major causes of differences in RGR, DM accumulation and grain yield related traits of wheat plants.

**Table 4:** Two-way ANOVA to show the significant effects of drought stress (DS), nitrogen (N) and their interaction on growth and yield attributes in wheat.

Factors	RGR	DM	GFR	GFD	GY	DI	NSpk/pot	G/spk	1000-grainWt
*F_Nitrogen_*	28.47^∗^	50.43^∗^	295.9^∗^	318.7^∗^	676.5^∗^	36.4^∗^	756.4^∗^	125.76^∗^	290.8^∗^
*F_Drought stress_*	83.8^∗^	89.5^∗^	223.3^∗^	148.3^∗^	114.6^∗^	1529.0^∗^	62.5^∗^	95.3^∗^	91.9^∗^
*F_Nx DS_*	3.14ns	1.4ns	18.37^∗^	5.85^∗^	3.83^∗^	4.49^∗^	4.82^∗^	4.32^∗^	13.85^∗^


*^∗^ and ns indicate significance at 0.05 level of probability. GFR, grain filling rate; GFD, grain filling duration; GY, grain yield/pot; DI, drought index; NSpk, number of spikes/pot; GSpk, grains/spike; DM, dry matter production and RGR, relative growth rate.*

Our results of no or slight yield reductions under moderate drought stress at vegetative stages were in agreement with the previous studies (Backhaus et al., 2014; Cui et al., 2015). Reduction in grain yield was attributed to reduced number of tillers under ST as well as to the limited number of grains/spike and reduced 1000-grain weight under SJ. The developmental retardation due to ST was significantly marked in the tillers rather than in the main stem. Under ST, most of the tillers came to death in the later stages reducing the net tillers with ears at maturity. Since during jointing stage the crop growth was rapid, and the most important part of photosynthetic reserves those are expected to contribute up to 57% of the final grain yield of wheat are assimilated during this stage (May, 1976). Therefore, unfavorable environmental conditions during this stage particularly decreased the physiological activities and grain yields. Water-stressed plants under N_0.24_ application showed a significantly higher grain yield as compared to N_0.16_. The same trend was also observed for WW plants, but the magnitude of increase in grain yield due to higher N application was more obvious under drought stress conditions rather than WW conditions. Comparatively, sustained growth and yield responses of wheat to drought stress at higher N application suggested that N supply could improve wheat response to drought stress.

## Conclusion

The results of this study showed the effects of drought stress and the significance of N availability on physiological activities, growth as well as grain yield in wheat. It was observed that combinations of drought stress during vegetative growth periods and N deficiency affected the responses of strongly interrelated physiological functions of wheat plants. An adequate N supply under drought stress enhanced Ψ_w_, MSI and antioxidant activity as well as reduced the downregulation of photosynthetic processes and penalties to grain yield. Along with physiological studies, molecular investigations are required for a better understanding of underlying mechanisms responsible for the N effect to enhance the wheat potential for the protective effect of N against drought stress. Since, undoubtedly to meet the future challenges of water limitations to agriculture would depend not only on breeding new varieties well adapted to water limitations, but also on the development of the cultural practices which in turn would enhance the crop potential for sustainable crop production.

## Author Contributions

MA, ZT, DJ, and TD conceived the idea and led the study design. MA carried out the experiment, performed analysis and wrote the paper. YC, YL, and RZ assisted in plant sampling and laboratory analysis. STA assisted manuscript writing and editing.

## Conflict of Interest Statement

The authors declare that the research was conducted in the absence of any commercial or financial relationships that could be construed as a potential conflict of interest.
